# Impact of bone-targeting agents on the clinical outcomes in patients with metastatic renal cell carcinoma treated with nivolumab: a sub-analysis of the Meet-URO 15 study

**DOI:** 10.3389/fonc.2026.1754327

**Published:** 2026-02-18

**Authors:** Francesco Pantano, Andrea Malgeri, Ugo De Giorgi, Marco Maruzzo, Giuseppe Fornarini, Paolo Zucali, Lucia Fratino, Michele Milella, Paolo Pedrazzoli, Giuseppe Procopio, Marco Stellato, Emanuele Naglieri, Hector Soto Parra, Marilena Di Napoli, Veronica Mollica, Marianna Tudini, Stefania Pipitone, Matteo Santoni, Riccardo Ricotta, Giuseppe Luigi Banna, Fabio Catalano, Alessia Cavo, Francesca Vignani, Cristina Masini, Chiara Casadei, Luca Galli, Franco Nolè, Mario Sorarù, Veronica Prati, Stefano Panni, Giandomenico Roviello, Giuseppe Prati, Franco Morelli, Carlo Messina, Francesco Atzori, Pasquale Rescigno, Daniele Santini, Sebastiano Buti, Sara Elena Rebuzzi

**Affiliations:** 1 Department of Medical Oncology, Fondazione Policlinico Universitario Campus Bio-Medico, Rome, Italy; 2 Medical Oncology Department, Istituto di ricovero e cura a carttere scientifico (IRCCS) Istituto Romagnolo per lo Studio dei Tumori (IRST) ‘Dino Amadori’, Meldola, Italy; 3 Oncologia 1, Istituto Oncologico Veneto, IOV - Istituto di ricovero e cura a carttere scientifico (IRCCS), Padova, Italy; 4 Medical Oncology Unit 1, Istituto di ricovero e cura a carttere scientifico (IRCCS) Ospedale Policlinico San Martino, Genoa, Italy; 5 Department of Oncology, Istituto di ricovero e cura a carttere scientifico (IRCCS), Humanitas Clinical and Research Center, Department of Biochemical Sciences, Humanitas University, Milano, Italy; 6 Department of Medical Oncology, CRO Aviano - Centro di Riferimento Oncologico Istituto di ricovero e cura a carttere scientifico (IRCCS), Aviano, Italy; 7 Department of Medical Oncology, Azienda Ospedaliera Universitaria Integrata di Verona, Verona, Italy; 8 Department of Internal Medicine and Medical Therapy, University of Pavia, Pavia, Italy; 9 Medical Oncology, Fondazione Istituto di ricovero e cura a carttere scientifico (IRCCS) - Istituto Nazionale dei Tumori, Milano, Italy; 10 Operative Unit (U.O.) Oncologia, Istituto di ricovero e cura a carttere scientifico (IRCCS) Istituto Tumori Giovanni Paolo II, Bari, Italy; 11 Department of Oncology, Medical Oncology, University Hospital Policlinico-San Marco, Catania, Italy; 12 Department of Urology and Gynecology, Istituto Nazionale Tumori Istituto di ricovero e cura a carttere scientifico (IRCCS) Fondazione G. Pascale, Napoli, Italy; 13 Medical Oncology, Istituto di ricovero e cura a carttere scientifico (IRCCS) - Azienda Ospedaliero-Universitaria di Bologna, Bologna, Italy; 14 Medical Oncology, Osp. San Salvatore, Azienda Sanitaria Locale 1 (ASL1) Avezzano Sulmona, L’Aquila, Italy; 15 Medical Oncology Unit, Department of Oncology and Hemathology, University Hospital of Modena, Modena, Italy; 16 Oncology Unit, Macerata Hospital, Macerata, Italy; 17 Oncology Unit, Istituto di ricovero e cura a carttere scientifico (IRCCS) MultiMedica, Sesto San Giovanni, Milano, Italy; 18 Department of Oncology, Portsmouth Hospitals University National Health Service (NHS) Trust, Portsmouth, United Kingdom; 19 Faculty of Science and Health, School of Pharmacy and Biomedical Sciences, University of Portsmouth, Portsmouth, United Kingdom; 20 Oncology Unit, Villa Scassi Hospital, Genoa, Italy; 21 Division of Medical Oncology, Ordine Mauriziano Hospital, Torino, Italy; 22 Azienda Unità Sanitaria Locale - Istituto di ricovero e cura a carttere scientifico (IRCCS) di Reggio Emilia, Reggio Emilia, Italy; 23 Medical Oncology Unit 2, Azienda Ospedaliera Universitaria Pisana, Pisa, Italy; 24 Medical Oncology Division of Urogenital and Head and Neck Tumors, Istituto Europeo Oncologico (IEO), European Institute of Oncology Istituto di ricovero e cura a carttere scientifico (IRCCS), Milano, Italy; 25 Medical Oncology, Ospedale Camposampiero, Padova, Italy; 26 Oncology Unit, Ospedale Michele e Pietro Ferrero, Verduno, Italy; 27 Medical Oncology Unit, Azienda Socio-Sanitaria Territoriale (ASST) - Istituti Ospitalieri Cremona Hospital, Cremona, Italy; 28 Department of Health Sciences, Section of Clinical Pharmacology and Oncology, University of Firenze, Firenze, Italy; 29 Medical Oncology Department, Casa Sollievo Della Sofferenza Hospital, Istituto di ricovero e cura a carttere scientifico (IRCCS), San Giovanni Rotondo, Italy; 30 Unità Operativa Complessa (UOC) Oncology, Fondazione Istituto San Raffaele Giglio di Cefalù, Cefalù, Italy; 31 Medical Oncology Department, University Hospital, University of Cagliari, Cagliari, Italy; 32 Translationsal and Clinical Research Institute, Centre for Cancer, Newcastle University, Newcastle Upon Tyne, United Kingdom; 33 Candiolo Cancer Institute, Fondazione Piemontese per la Ricerca sul Cancro - IRCCS (FPO-IRCCS), Candiolo, Italy; 34 Oncology A, Department of Hematology, Oncology and Dermatology, AOU Policlinico Umberto I, Rome, Italy; 35 Department of Medico-Surgical Sciences and Biotechnologies, Sapienza University of Rome, Rome, Italy; 36 Medical Oncology Unit, University Hospital of Parma, Parma, Italy; 37 Medical Oncology Unit, Ospedale San Paolo, Savona, Italy; 38 Department of Internal Medicine and Medical Specialties (Di.M.I.), University of Genoa, Genoa, Italy

**Keywords:** bone target agents, denosumab, immuno-checkpoint inhibitors, nivolulmab, renal cell carcinoma

## Abstract

**Background:**

Immune checkpoint inhibitors have revolutionized the treatment landscape for metastatic renal cell carcinoma (mRCC). However, some patients fail to experience durable benefits, especially those with bone metastases.

**Objective:**

This study aimed to evaluate the impact of bone-targeting agents (BTAs), specifically denosumab and zoledronic acid (ZA), on the clinical outcomes of patients with mRCC treated with nivolumab.

**Methods:**

This retrospective study analyzed data from the Meet-URO 15 trial on patients with mRCC who received nivolumab, categorizing them into BTA and non-BTA groups. Survival outcomes were assessed, with inverse probability of treatment weighting (IPTW) adjustment for confounding variables. Subsequently, the specific impact of different BTAs on the clinical outcomes was explored.

**Results:**

Of 203 mRCC patients with bone metastases, 38 received BTAs (BTA group) while 138 did not (non-BTA group). BTA treatment significantly improved the median progression-free survival (PFS) (291 *vs*. 117 days, *p* = 0.005) and overall survival (OS) (960 *vs*. 397 days, *p* = 0.008) compared with the non-BTA group, with a reduced risk of death (HR = 0.57, 95%CI = 0.34–0.95, *p* = 0.031) and progression or death (HR = 0.57, 95%CI = 0.35–0.92, *p* = 0.023) at multivariate analyses. IPTW adjustment confirmed these survival benefits, with a reduced risk of death (HR = 0.55–95%CI = 0.39–0.76, *p* < 0.001) and progression or death (HR = 0.58, 95%CI = 0.42–0.79, *p* < 0.001) in BTA patients. Furthermore, denosumab, compared with ZA and the non-BTA group, demonstrated superior OS (1,662 *vs*. 681 *vs*. 411 days, *p* < 0.001) and PFS (1,101 *vs*. 242 *vs*. 132 days, *p* < 0.001) in the same IPTW-adjusted population.

**Conclusion:**

This study suggests a potential beneficial impact of BTAs, especially denosumab, on the clinical outcomes after nivolumab therapy in mRCC patients with bone metastases. Prospective trials are needed to better define the impact of BTAs in these patients.

## Background

Immune checkpoint inhibitors (ICIs) play a crucial role in the armamentarium of metastatic renal cell carcinoma (mRCC), demonstrating their effect both as a single agent and in combination with anti-vascular endothelial growth factor tyrosine kinase inhibitors (VEGF-TKIs). Nivolumab is the first ICI approved for patients with mRCC after the positive results of the Checkmate 025 trial that assessed its survival superiority over everolimus after at least a precedent line with anti-VEGF TKI (1). Nevertheless, there is a proportion of patients with renal cell carcinoma (RCC) that does not obtain a remarkable and durable benefit from immunotherapies.

Bone metastases occur approximately from 30% to 50% of RCCs and represent an established negative prognostic factor in this malignancy, associated with dismal survival and inferior response to treatments (2–5). Bone metastases in mRCC are primarily osteolytic and are correlated with an augmented risk of skeletal-related events (SREs) through a complex process resulting in decreased bone integrity. SREs are defined as pathological fracture, radiotherapy to bone, surgery to bone, spinal cord compression, and hypercalcemia (6).

Therefore, the existence of bone metastases poses a formidable challenge in the treatment of these patients, given its well-known association with a bleak prognosis. Findings from the multicentric prospective phase II GETUG-AFU-26 NIVOREN trial revealed the reduced efficacy of nivolumab treatment in mRCC patients with bone metastases when compared to those without (7).

Bone-targeting agents (BTAs), such as bisphosphonates and denosumab, a fully human monoclonal antibody against receptor activator of nuclear factor kappa-β ligand (RANKL), are commonly administered in solid tumors with bone metastases, including mRCC, to prevent SREs (5, 7, 8). Despite their utility in decreasing the occurrence of SREs, there are few and controversial data on the impact of BTAs on the survival outcomes of patients with mRCC (9).

In recent years, increasing attention has been directed toward the interaction between the bone microenvironment and the antitumor immune responses, particularly in the context of immune checkpoint inhibition. Beyond their established role in the prevention of SREs, BTAs may influence immune regulation within the bone niche through modulation of osteoclast activity and the RANK–RANKL signaling pathway. Emerging clinical and translational evidence suggests that the activation of the RANK–RANKL axis may negatively affect the response to ICIs in mRCC, positioning RANKL as a potential biomarker for resistance to immunotherapy (10, 11).

On this basis, we hypothesized that exposure to BTAs could be associated with improved clinical outcomes in mRCC patients with bone metastases treated with nivolumab. Using a large, multicenter real-world cohort from the Meet-URO 15 study, which investigated 571 patients with mRCC who received nivolumab in the second-line (or higher) setting from October 2015 to November 2019, the present analysis was designed to explore whether the use of BTAs is associated with differences in the progression-free survival (PFS) and overall survival (OS) of these patients (12–14).

## Materials and methods

### Study design

This study is a *post-hoc* analysis of the retrospective, multicenter Meet-URO 15 study, conducted to explore the association between exposure to BTAs and the clinical outcomes of patients with mRCC presenting with bone metastases and treated with nivolumab in routine clinical practice.

### Patient cohort

All patients enrolled in the Meet-URO 15 study who had documented bone involvement at baseline were considered for inclusion. Within this population, patients were stratified according to the use of BTAs into those who received BTAs (BTA group) and those who did not (non-BTA group). Bone-targeted therapy was defined as treatment with either denosumab or zoledronic acid.

To limit potential immortal time bias, only those patients who initiated BTA therapy before starting nivolumab or before the first radiological reassessment following nivolumab initiation were included in the BTA group, while patients who started BTAs after the first disease evaluation were excluded. Clinical, pathological, and treatment-related data were retrospectively collected from medical records across participating centers, reflecting real-world clinical practice.

### Treatments

Nivolumab was administered intravenously according to approved schedules, initially at a weight-based dose of 3 mg/kg every 2 weeks and, from May 2018 onward, at a fixed dose of 240 mg every 2 weeks or 480 mg every 4 weeks, depending on local practice. Treatment was continued until disease progression, unacceptable toxicity, death, or patient decision. Zoledronic acid and denosumab were administered according to standard dosing regimens, with zoledronic acid given intravenously at 4 mg every 4 weeks and denosumab administered subcutaneously at 120 mg every 4 weeks. Both agents were continued until unacceptable toxicity, death, or treatment discontinuation.

### Outcome variables

The primary objective of the study was to explore the association between BTA exposure and the clinical outcomes of mRCC patients with bone metastases treated with nivolumab.

OS was defined as the interval between nivolumab initiation and death from any cause, whereas PFS was defined as the time from nivolumab initiation to radiological or clinical disease progression or death, whichever occurred first. Patients without documented events were censored at the date of the last follow-up.

### Statistical analysis

Survival outcomes were estimated using the Kaplan–Meier method and compared between groups using the log-rank test. Cox proportional hazards regression models were employed to evaluate the association between BTA exposure and survival outcomes, both in univariate analysis and after adjustment for clinically relevant covariates. The variables included into the multivariable models were selected *a priori* based on their established prognostic significance in mRCC and included age, sex, prior nephrectomy, metastatic disease at diagnosis, the International Metastatic RCC Database Consortium (IMDC) risk category, histology, treatment line, visceral metastases, and the neutrophil-to-lymphocyte ratio (NLR), categorized into high (>3.2) and low (≤3.2).

Given the retrospective nature of the study and the non-randomized allocation of the BTA therapy, the potential for selection bias and confounding was explicitly addressed. In addition to restricting BTA exposure to the pre-first reassessment period to reduce immortal time bias, multivariable adjustment for known prognostic factors was performed. Furthermore, inverse probability of treatment weighting (IPTW) was applied to balance the observed baseline characteristics between treatment groups, thereby approximating a pseudo-randomized population. Covariate balance after weighting was assessed using standardized mean differences, and all survival analyses were subsequently repeated in the weighted cohort. Exploratory analyses evaluating the outcomes according to the specific BTA administered were conducted within the IPTW-adjusted population.

All statistical analyses were two-sided, and statistical significance was defined as a *p*-value less than 0.05. Analyses were performed using R software.

## Results

### Patient characteristics

Among the 571 patients with mRCC treated with nivolumab included in the Meet-URO 15 study, 203 patients had bone metastases at baseline. Of these, 65 patients received BTAs; however, 27 were excluded as the BTA treatment was initiated after the first radiological restaging. Consequently, 38 patients constituted the BTA group, while 138 patients with bone metastases who did not receive BTAs formed the non-BTA group.

The baseline clinicopathological characteristics of the two groups are reported in **Tables 1A**, **B**. Notably, the two populations differed with respect to the presence of metastatic disease at diagnosis (68.4% in the BTA group *vs*. 47.1% in the non-BTA group; *p* = 0.032) and the IMDC risk distribution. Patients in the BTA group more frequently belonged to the favorable-risk category (36.8% *vs*. 8.0%), whereas intermediate- and poor-risk categories were more prevalent in the non-BTA group (*p* < 0.001).

**Table 1:** Patients baseline characteristics according bone target agents use in unadjusted population (A) and in Inverse probability of treatment weighting (IPTW) adjusted population (B).

A					
Clinical characteristics		Bone target agents		p-value
		No	Yes	
n		138	38	
Sex (%)	Female	43 (31.2)	13 (34.2)	0.872
	Male	95 (68.8)	25 (65.8)	
Age	Median (IQR)	74.9 ± 4.9	74.7 ± 4.0	0.718
Nephrectomy (%)	No	24 (17.4)	4 (10.5)	0.439
	Yes	114 (82.6)	34 (89.5)	
Metastatic at Diagnosis (%)	No	65 (47.1)	26 (68.4)	**0.032**
	Yes	73 (52.9)	12 (31.6)	
Histology (%)	ClearCell	123 (89.1)	34 (89.5)	1
	Other	15 (10.9)	4 (10.5)	
Treatment Line		2.4 ± 0.8	2.6 ± 0.8	0.22
IMDC Score (%)	1	11 (8.0)	14 (36.8)	**<0.001**
	2	96 (69.6)	18 (47.4)	
	3	31 (22.5)	6 (15.8)	
Visceral Disease (%)	No	19 (13.8)	8 (21.1)	0.392
	Yes	119 (86.2)	30 (78.9)	
NLR (%)	High (> 3.2)	48 (43.2)	18 (54.5)	0.345
	Low (< 3.2)	63 (56.8)	15 (45.5)	

Values shown in bold indicate statistically significant results.

**Table:** 

B					
Clinical characteristics		Bone target agents		Total	p-value
		Yes	No		
Sex (%)	Female	55 (34.0)	58 (43.6)	113 (38.3)	0.115
	Male	107 (66.0)	75 (56.4)	182 (61.7)	
Age	Median (IQR)	75.9 (75.1 to 76.7)	75.9 (74.8 to 76.5)	75.9 (75.0 to 76.6)	0.762
Nephrectomy (%)	No	26 (16.0)	23 (17.3)	49 (16.6)	0.898
	Yes	136 (84.0)	110 (82.7)	246 (83.4)	
Metastatic at diagnosis (%)	No	82 (50.6)	65 (48.9)	147 (49.8)	0.856
	Yes	80 (49.4)	68 (51.1)	148 (50.2)	
Histology (%)	Clear Cell	146 (90.1)	122 (91.7)	268 (90.8)	0.785
	Other	16 (9.9)	11 (8.3)	27 (9.2)	
Treatment line	Median (IQR)	2.0 ± 1.0	2.0 ± 1.0	2.0 ± 1.0	0.148
IMDC score (%)	1	18 (11.1)	17 (12.8)	35 (11.9)	0.267
	2	109 (67.3)	97 (72.9)	206 (69.8)	
	3	35 (21.6)	19 (14.3)	54 (18.3)	
Visceral disease (%)	No	24 (14.8)	20 (15.0)	44 (14.9)	1
	Yes	138 (85.2)	113 (85.0)	251 (85.1)	
NLR (%)	High (> 3.2)	55 (42.0)	54 (51.9)	109 (46.4)	0.166
	Low (< 3.2)	76 (58.0)	50 (48.1)	126 (53.6)	

IMDC, International Metastatic RCC Database Consortium; NLR, neutrophil-to-lymphocyte ratio; IQR, interquartile range. Values shown in bold indicate statistically significant results

After IPTW adjustment, all baseline characteristics were well balanced between the two groups (**Table 1B**). The median follow-up for the overall population was 58.5 months (95%CI = 53.1–66.7).

### Survival outcomes according to the BTA group

**Figure 1:**
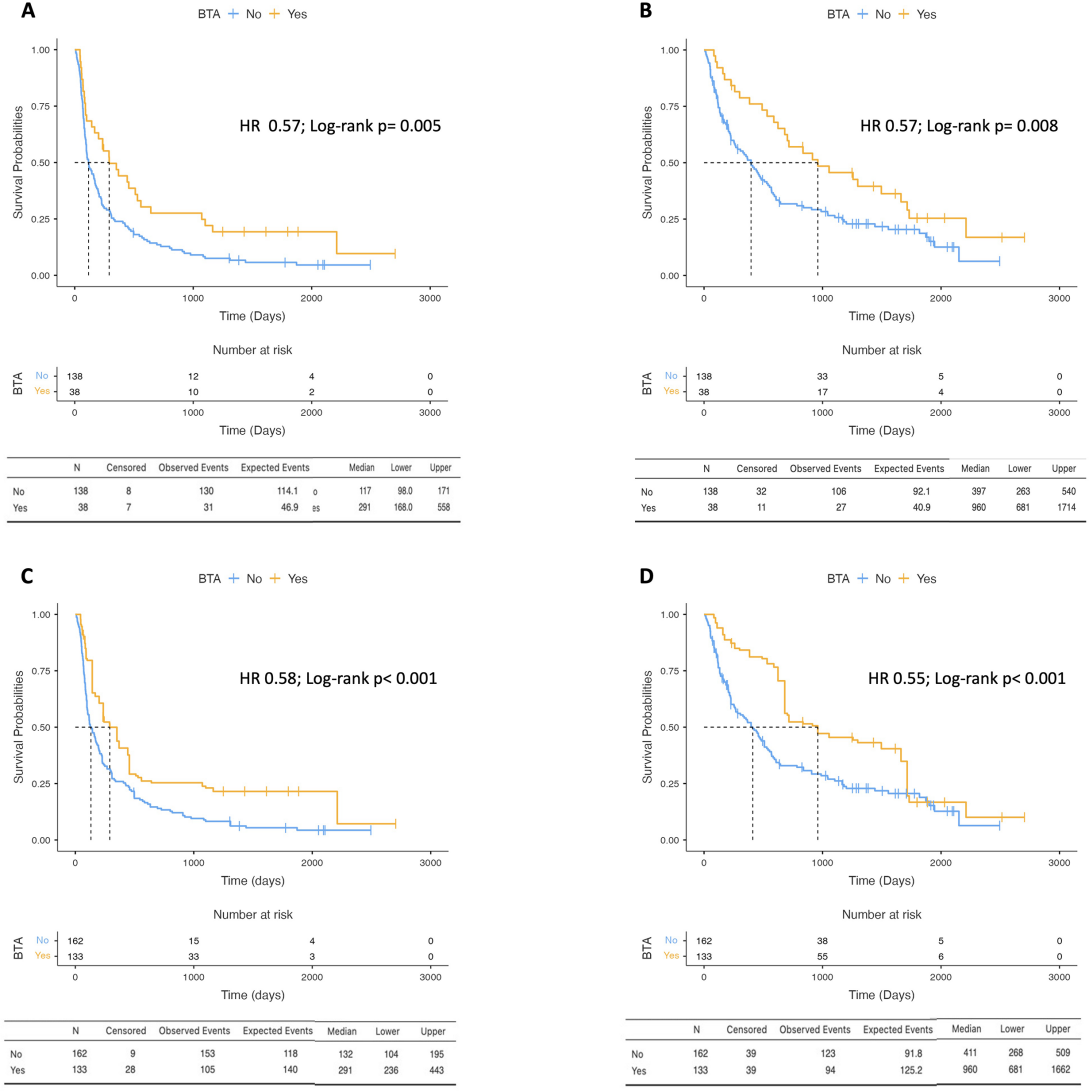
Kaplan-Meier curves reporting the progression-free survival **(A)** and overall survival **(B)** in patients who received Bone Target agents (BTA-group) and in non BTA-group in unadjusted population. Kaplan-Meier curves reporting the progression-free survival **(C)** and overall survival **(D)** in patients who received Bone Target agents (BTA-group) and in non BTA-group in Inverse probability of treatment weighting (IPTW) adjusted population.

Patients in the BTA group showed a significantly higher median PFS (mPFS) compared with patients in the non-BTA group, respectively 291 days (95%CI = 168–558) *vs*. 117 days (95%CI = 98–171; *p* = 0.005) (**Figure 1A**). Similarly, the median OS (mOS) was much longer in patients undergoing BTA treatment compared with those who did not, respectively 960 days (95%CI = 681–1,714) *vs*. 397 days (95%CI = 263–540; *p* = 0.008) (**Figure 1B**). Univariate analysis was performed for PFS and OS, including all clinicopathological variables (**Table 2A**). Clinically relevant factors, including sex, age, histology, prior nephrectomy, and the presence of baseline metastasis, showed no significant associations with the studied survival parameters, while a high NLR (>3.2) and the IMDC score did. The benefit of BTA treatment on the survival outcomes was confirmed in a multivariate analysis adjusted for the aforementioned factors, with a 43% reduction in the risk of death (HR = 0.57, 95%CI = 0.34–0.95, *p* = 0.031) and a 43% reduction in the risk of progression or death (HR = 0.57, 95%CI = 0.35–0.92, *p* = 0.023) (**Table 2B**). Moreover, after IPTW adjustment to balance the different covariates in the two groups, exposure to BTA treatment demonstrated advantages in the survival outcomes, with a mOS of 960 days (95%CI = 681–1662) in the BTA group *vs*. 411 days (95%CI = 266–509) in the non-BTA group (*p* < 0.001) and a mPFS of 291 days (95%CI = 236–443) *vs*. 132 days, respectively (95%CI = 104–195; *p* < 0.001) (**Figures 1C, D**). The univariate analysis for each individual clinicopathological factor and the multivariate analysis adjusted for the same factors also confirmed a reduction in the risk of death (HR = 0.55, 95%CI = 0.39–0.76, *p* < 0.001) and the risk of progression or death (HR = 0.58, 95%CI = 0.42–0.79, *p* = 0.001) in the BTA group in the IPTW-adjusted population (**Tables 2C, D**).

**Table 2:** Univariate and multivariate cox regression analysis for progression-free survival (A) and overall survival (B) in unadjusted population.

A			
Clinical characteristics	Parameter	HR univariable (95% CI, p value)	HR multivariable (95% CI, p value)
Sex	Female	–	–
	Male	0.74 (0.49-1.11, p = 0.147)	0.68 (0.44-1.05, p = 0.078)
Nephrectomy	No	–	–
	Yes	0.62 (0.37-1.03, p = 0.067)	0.85 (0.46-1.59, p = 0.618)
Metastatic at Diagnosis	No	–	–
	Yes	1.19 (0.82-1.74, p = 0.353)	0.76 (0.48-1.22, p = 0.258)
Histology	ClearCell	–	–
	Other	0.99 (0.56-1.78, p = 0.985)	0.75 (0.41-1.41, p = 0.376)
Treatment Line	2	–	–
	>2	0.81 (0.54-1.20, p = 0.292)	0.84 (0.55-1.30, p = 0.437)
IMDC Score	1	–	–
	2	**1.99 (1.09-3.63, p = 0.025)**	1.54 (0.79-3.02, p = 0.205)
	3	**3.82 (1.95-7.48, p < 0.001)**	**3.39 (1.56-7.34, p = 0.002)**
NLR	High	–	–
	Low	**0.53 (0.36-0.77, p = 0.001)**	**0.52 (0.34-0.79, p = 0.002)**
BTA	No	–	–
	Yes	**0.54 (0.34-0.86, p = 0.009)**	**0.57 (0.34-0.95, p = 0.031)**
Age	Mean (SD)	0.98 (0.94-1.02, p = 0.243)	1.01 (0.97-1.05, p = 0.640)

Univariate and Multivariate Cox regression analysis for Progression-Free Survival (C) and Overall Survival (D) in Inverse probability of treatment weighting (IPTW) adjusted population.

Values shown in bold indicate statistically significant results.

**Table:** 

B			
Clinical characteristics	Parameter	HR univariable (95% CI, p value)	HR multivariable (95% CI, p value)
Sex	Female	–	–
	Male	0.74 (0.49-1.11, p = 0.147)	0.68 (0.44-1.05, p = 0.078)
Nephrectomy	No	–	–
	Yes	0.62 (0.37-1.03, p = 0.067)	0.85 (0.46-1.59, p = 0.618)
Metastatic at Diagnosis	No	–	–
	Yes	1.19 (0.82-1.74, p = 0.353)	0.76 (0.48-1.22, p = 0.258)
Histology	Clear Cell	–	–
	Other	0.99 (0.56-1.78, p = 0.985)	0.75 (0.41-1.41, p = 0.376)
Treatment Line	2	–	–
	> 2	0.81 (0.54-1.20, p = 0.292)	0.84 (0.55-1.30, p = 0.437)
IMDC Score	1	–	–
	2	**1.99 (1.09-3.63, p = 0.025)**	1.54 (0.79-3.02, p = 0.205)
	3	**3.82 (1.95-7.48, p < 0.001)**	**3.39 (1.56-7.34, p = 0.002)**
NLR	High	–	–
	Low	**0.53 (0.36-0.77, p = 0.001)**	**0.52 (0.34-0.79, p = 0.002)**
BTA	No	–	–
	Yes	**0.54 (0.34-0.86, p = 0.009)**	**0.57 (0.34-0.95, p = 0.031)**
Age	Mean (SD)	0.98 (0.94-1.02, p = 0.243)	1.01 (0.97-1.05, p = 0.640)

Values shown in bold indicate statistically significant results.

**Table:** 

C			
Clinical covariate	Parameter	HR univariable (95% CI, p value)	HR multivariable (95% CI, p value)
Sex	Female	–	–
	Male	1.05 (0.77-1.43, p = 0.758)	0.71 (0.50-1.00, p = 0.050)
Nephrectomy	No	–	–
	Yes	**2.07 (1.34-3.18, p = 0.001)**	1.57 (0.93-2.66, p = 0.095)
Metastatic at Diagnosis	No	–	–
	Yes	0.91 (0.69-1.21, p = 0.533)	0.78 (0.55-1.12, p = 0.176)
Histology	ClearCell	–	–
	Other	0.76 (0.47-1.22, p = 0.249)	**0.57 (0.33-0.98, p = 0.041)**
IMDC Score	1	–	–
	2	1.53 (0.98-2.40, p = 0.063)	1.24 (0.76-2.02, p = 0.388)
	3	**1.69 (1.00-2.86, p = 0.049)**	**1.89 (1.07-3.32, p = 0.027)**
Visceral Disease	No	–	–
	Yes	**1.96 (1.29-2.97, p = 0.001)**	**2.08 (1.33-3.26, p = 0.001)**
NLR	High	–	–
	Low	**0.54 (0.41-0.72, p < 0.001)**	**0.52 (0.38-0.71, p < 0.001)**
BTA	No	–	–
	Yes	**0.49 (0.37-0.66, p < 0.001)**	**0.58 (0.42-0.79, p = 0.001)**
Age	Mean (SD)	**0.97 (0.94-1.00, p = 0.048)**	1.01 (0.98-1.04, p = 0.538)
Treatment Line	Mean (SD)	**0.75 (0.61-0.93, p = 0.009)**	**0.77 (0.62-0.95, p = 0.016)**

Values shown in bold indicate statistically significant results.

**Table:** 

D			
Clinical covariate	Parameter	HR univariable (95% CI, p value)	HR multivariable (95% CI, p value)
Sex	Female	–	–
	Male	0.75 (0.54-1.04, p = 0.085)	**0.55 (0.39-0.79, p = 0.001)**
Nephrectomy	No	–	–
	Yes	1.44 (0.91-2.28, p = 0.116)	1.18 (0.69-2.02, p = 0.548)
Metastatic at Diagnosis	No	–	–
	Yes	0.91 (0.67-1.24, p = 0.553)	**0.54 (0.36-0.80, p = 0.002)**
Histology	ClearCell	–	–
	Other	0.86 (0.52-1.41, p = 0.550)	0.69 (0.40-1.20, p = 0.193)
IMDC Score	1	–	–
	2	**1.81 (1.08-3.03, p = 0.025)**	1.68 (0.97-2.90, p = 0.064)
	3	**2.85 (1.59-5.09, p < 0.001)**	**4.21 (2.22-7.99, p < 0.001)**
Visceral Disease	No	–	–
	Yes	**2.03 (1.27-3.24, p = 0.003)**	**2.59 (1.55-4.32, p < 0.001)**
NLR	High	–	–
	Low	**0.53 (0.39-0.72, p < 0.001)**	**0.48 (0.34-0.67, p < 0.001)**
BTA	No	–	–
	Yes	**0.48 (0.35-0.66, p < 0.001)**	**0.55 (0.39-0.76, p < 0.001)**
Age	Mean (SD)	0.97 (0.94-1.00, p = 0.059)	1.01 (0.97-1.05, p = 0.639)
Treatment Line	Mean (SD)	0.81 (0.64-1.01, p = 0.064)	0.84 (0.67-1.05, p = 0.128)

IMDC, International Metastatic RCC Database Consortium; NLR, neutrophil-to-lymphocyte ratio; BTA, bone target agent; SD, standard deviation.

Values shown in bold indicate statistically significant results.

Patients treated with BTAs experienced longer PFS compared with those who did not receive BTAs. The mPFS was 291 days (95%CI = 168–558) in the BTA group *vs*. 117 days (95%CI = 98–171) in the non-BTA group (*p* = 0.005) (**Figure 1A**). Similarly, the mOS was longer in the BTA group (960 days; 95%CI = 681–1,714) compared with the non-BTA group (397 days; 95%CI = 263–540, *p* = 0.008) (**Figure 1B**).

The univariate analysis confirmed the association of higher NLR and IMDC risk score with poorer PFS and OS, while the other clinicopathological variables did not show statistically significant associations (**Table 2A**). In the multivariable models adjusted for clinically relevant prognostic factors, BTA exposure remained associated with a lower risk of death (HR = 0.57, 95%CI = 0.34–0.95, *p* = 0.031) and progression or death (HR = 0.57, 95%CI = 0.35–0.92, *p* = 0.023) (**Table 2B**).

These findings were consistent after IPTW adjustment. In the weighted population, the mOS was 960 days (95%CI = 681–1,662) in the BTA group *vs*. 411 days (95%CI = 266–509) in the non-BTA group (*p* < 0.001), while the mPFS was 291 days (95%CI = 236–443) *vs*. 132 days (95%CI = 104–195), respectively (*p* < 0.001) (**Figures 1C, D**). Multivariable analyses in the IPTW-adjusted cohort confirmed an association between BTA exposure and reduced risk of death and progression or death (**Tables 2C, D**).

### Survival outcomes according to the specific BTA

**Figure 2:**
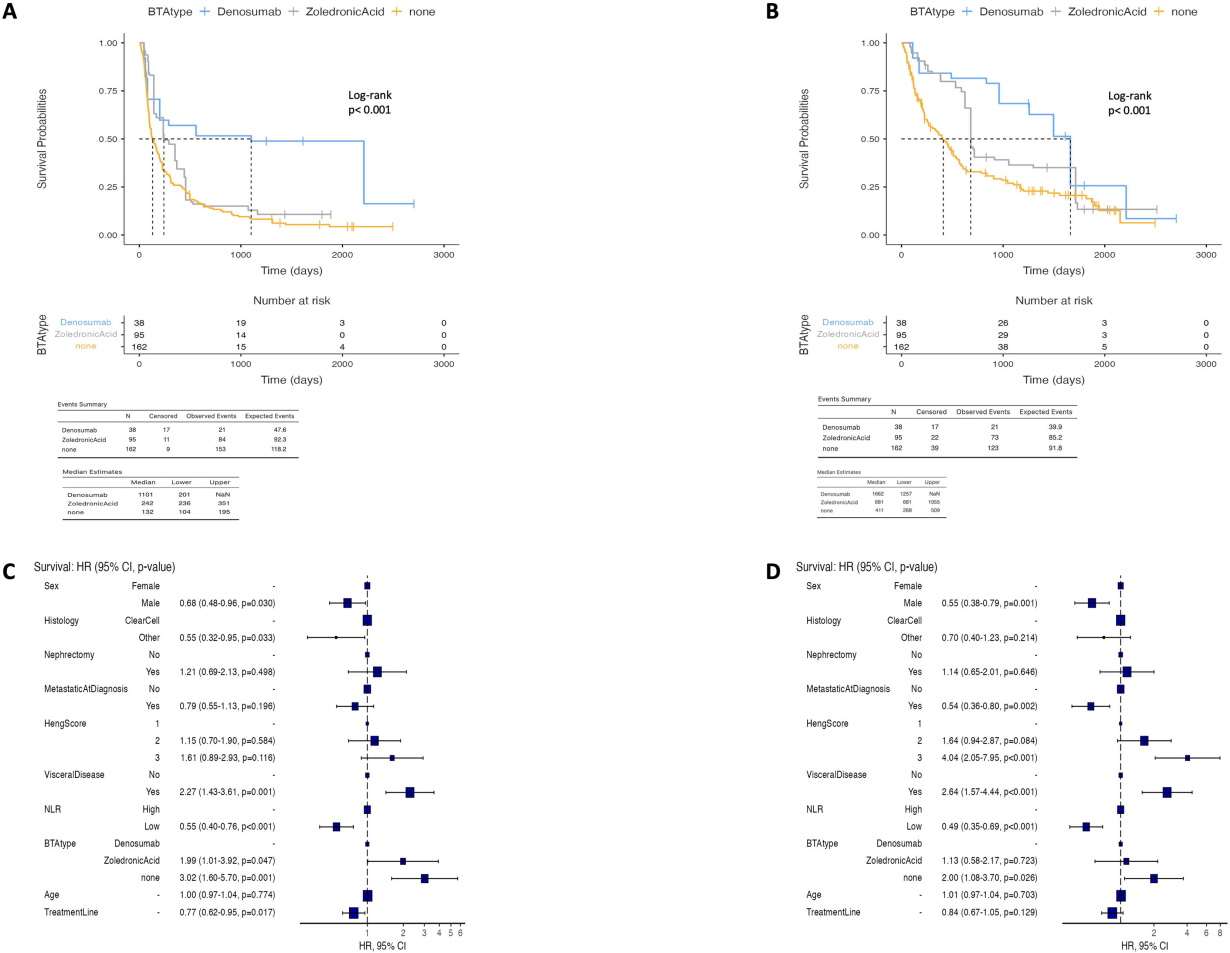
Kaplan-Meier curves reporting the progression-free survival **(A)** and the overall survival **(B)** according Bone target agent type use in Inverse probability of treatment weighting (IPTW) adjusted population. Multivariate Cox regression analysis for progression-free survival **(C)** and overall survival **(D)**.

In the exploratory analyses within the same IPTW-adjusted population, the outcomes were further evaluated according to the specific BTA administered. Patients treated with denosumab (*n* = 38) showed longer OS and PFS compared with both the zoledronic acid (*n* = 95) and non-BTA groups (*n* = 162). The mOS was 1,662 days (95%CI = 1,257–not reached) in the denosumab group compared with 681 days (95%CI = 681–1,055) in the zoledronic acid group and 411 days (95%CI = 266–509) in the non-BTA group (**Figure 2B**). The mPFS followed a similar pattern (**Figure 2A**).

In the multivariable analysis, lack of BTA exposure was associated with an increased risk of death and progression compared with denosumab, while zoledronic acid was associated with a higher risk of progression or death compared with denosumab, although the difference in OS did not reach statistical significance (**Figures 2C, D**).

## Discussion

Our analysis of a real-world, multicenter cohort of mRCC patients with bone metastases treated with nivolumab suggests a favorable efficacy profile of exposure to BTAs. These patterns were consistently observed across multivariable analyses and remained evident after IPTW, suggesting that the observed differences were not solely driven by imbalances in the baseline prognostic factors. Nevertheless, given the retrospective and non-randomized nature of the study, these findings should be interpreted as hypothesis-generating rather than indicative of a causal relationship.

Bone metastases represent a well-established negative prognostic factor in mRCC, reflecting both advanced disease biology and the unique immunosuppressive features of the bone microenvironment. Clinical evidence from the GETUG-AFU-26 NIVOREN trial has shown reduced efficacy of nivolumab in patients with bone involvement, underscoring the unmet need for strategies capable of mitigating the adverse impact of skeletal disease on the immunotherapy outcomes (7). In this context, our findings suggest that modulation of the bone microenvironment through BTAs may be associated with more favorable clinical outcomes in this patient population treated with ICIs.

From a biological perspective, increasing evidence supports a complex interplay between bone remodeling, immune regulation, and tumor progression. The bone marrow niche is enriched in immunosuppressive cellular populations, including regulatory T cells and myeloid-derived suppressor cells, which may limit effective antitumor immune responses. In addition, tumor-driven osteoclast activation and bone resorption contribute to the release of immunosuppressive cytokines such as interleukin-6 and transforming growth factor beta, further reinforcing an immune-tolerant microenvironment. Through inhibition of osteoclast-mediated bone resorption, BTAs may indirectly modulate these processes, potentially influencing the immune cell composition and function within the bone niche (15–18).

Recent translational data have provided additional support for the relevance of the RANK–RANKL axis in this setting. Elevated baseline circulating RANKL levels have been associated with poorer survival and reduced response to nivolumab in patients with mRCC, suggesting that the activation of this pathway may represent a mechanism of resistance to immune checkpoint inhibition (10).

Our exploratory analyses suggest that denosumab exposure was associated with more favorable survival outcomes compared with zoledronic acid and no BTA exposure. While these observations should be interpreted with caution due to the limited sample size and potential residual confounding, they are consistent with the hypothesis that direct inhibition of RANKL may exert a more pronounced modulation of the bone–immune interface than indirect anti-resorptive mechanisms. Preclinical studies have shown that RANKL blockade alone may have limited antitumor activity, but can enhance the efficacy of ICIs when used in combination, potentially through effects on immune tolerance and tumor microenvironment (TME) remodeling. However, the exact mechanisms underlying these interactions remain incompletely understood.

The possible synergistic mechanisms between BTA, especially denosumab, and ICI remain unclear.

Few studies in mouse cancer models have explored how the inhibition of RANKL affects antitumor immunity *in vivo*, with limited efficacy observed in RANKL inhibition alone for controlling tumor growth in melanoma, colon carcinoma, and prostate carcinoma models. Enhanced tumor control was observed when combining RANKL inhibition with immunotherapy, possibly due to the cross-modulation of the TME and the interruption of local immunosuppressive pathways mediated by RANK-expressing immune cells in the TME. Another potential mechanism involves the interruption of thymic central tolerance (11, 19–21).

In the literature, other studies have explored possible additive effects between BTAs, in particular anti-RANKL agents, and ICIs in other tumors. The initial evidence includes two case reports of patients with aggressive melanoma treated with anti-CTLA4 and denosumab, who experienced substantial and prolonged disease control. Furthermore, a retrospective analysis of US electronic health record data in patients with melanoma and non-small cell lung cancer (NSCLC) with bone metastases demonstrated that the concurrent combination of ICI and denosumab could enhance the antitumor response and improve outcomes (22–25).

It is noteworthy that zoledronic acid, although primarily classified as a bisphosphonate, has also been shown to interfere with the RANK–RANKL pathway and to exert immunomodulatory effects, including the activation of γδ T cells. Therefore, the differences observed between denosumab and zoledronic acid in this study should not be interpreted as definitive evidence of superiority, but rather as an indication of potentially distinct biological effects that merit further investigation in prospective studies (26, 27).

Several limitations of this analysis must be acknowledged. Firstly, the retrospective design precludes control over treatment allocation and introduces the possibility of residual confounding despite multivariable adjustment and IPTW correction. Secondly, patients with longer survival or less aggressive disease may have been more likely to receive BTAs, potentially introducing survivorship bias; however, exclusion of patients initiating BTA therapy after the first radiological reassessment was implemented to mitigate immortal time bias. Thirdly, heterogeneity in the BTA type, timing, and duration, as well as in prior and subsequent systemic therapies, may have influenced the outcomes and cannot be fully accounted for in this analysis. Another important limitation of the present analysis is that the potential dose–time effect of BTAs could not be adequately assessed. Information regarding treatment duration, cumulative exposure, and adherence was heterogeneous and not uniformly available across centers, precluding a formal evaluation of whether the magnitude of the observed effect varied according to treatment intensity or duration. As a consequence, the present findings do not allow conclusions with regard to an optimal timing or duration of BTA therapy in this setting. Moreover, the safety outcomes related to BTAs, including medication-related osteonecrosis of the jaw (MRONJ), were not systematically collected in the Meet-URO 15 dataset and could therefore not be formally analyzed. Therefore, the potential clinical benefit suggested by the present findings should always be weighed against the known toxicity profile of BTAs, and careful patient selection and monitoring remain essential in routine practice. Finally, mechanistic correlates, such as circulating RANKL levels or immune profiling of the bone microenvironment, were not available and would be necessary to strengthen biological interpretations.

Despite these limitations, the present study provides real-world evidence supporting an association between BTA exposure and improved clinical outcomes in mRCC patients with bone metastases treated with nivolumab. These findings align with emerging translational data implicating the RANK–RANKL axis in the modulation of the response to immune checkpoint inhibition and support further prospective evaluation of combined strategies targeting both tumor immunity and the bone microenvironment. Ongoing clinical trials, such as KEYPAD, will be crucial to clarify the clinical relevance and mechanistic basis of these observations (28).

## Conclusions

In conclusion, our study suggests that BTA exposure is associated with more favorable survival outcomes among patients with mRCC treated with nivolumab. The observed positive impact on the survival outcomes, even after performing an IPTW adjustment to balance the clinicopathological factors in our populations, suggests the potential role of BTAs in optimizing the effectiveness of nivolumab therapy. Additional prospective studies are necessary to validate our findings, in particular in patients eligible for immune oncology–tyrosine kinase inhibitor (IO–TKI) and IO–IO combination therapies.

## Data Availability

The raw data supporting the conclusions of this article will be made available by the authors, without undue reservation.
